# Transcriptome-wide m^6^A methylome during osteogenic differentiation of human adipose-derived stem cells

**DOI:** 10.1186/s13287-021-02508-1

**Published:** 2021-09-01

**Authors:** Wentian Sun, Yidan Song, Kai Xia, Liyuan Yu, Xinqi Huang, Zhihe Zhao, Jun Liu

**Affiliations:** 1 State Key Laboratory of Oral Diseases & National Clinical Research Center for Oral Diseases, West China Hospital of Stomatology, Sichuan University, No. 14, 3rd Section, South Renmin Road, Chengdu, 610041 Sichuan China; 2 Department of Orthodontics, West China Hospital of Stomatology, Sichuan University, No. 14, 3rd Section, South Renmin Road, Chengdu, 610041 Sichuan China

**Keywords:** Adipose-derived stem cell, m^6^A, MeRIP-seq, N^6^-methyladenosine, Osteogenic differentiation, RNA-seq

## Abstract

**Objectives:**

Adipose-derived stem cells are frequently used for bone regeneration both in vitro and in vivo. N^6^-methyladenosine (m^6^A) is the most abundant post-transcriptional modification on eukaryotic RNAs and plays multifaceted roles in development and diseases. However, the regulatory mechanisms of m^6^A in osteogenic differentiation of human adipose-derived stem cells (hASCs) remain elusive. The present study aimed to build the transcriptome-wide m^6^A methylome during the osteogenic differentiation of hASCs.

**Materials and methods:**

hASCs were harvested after being cultured in a basic or osteogenic medium for 7 days, and the osteogenic differentiation was validated by alkaline phosphatase (ALP) and Alizarin Red S staining, ALP activity assay, and qRT-PCR analysis of ALP, RUNX2, BGLAP, SPP1, SP7, and COL1A1 genes. The m^6^A level was colorimetrically measured, and the expression of m^6^A regulators was confirmed by qRT-PCR and western blot. Moreover, m^6^A MeRIP-seq and RNA-seq were performed to build the transcriptome and m^6^A methylome. Furthermore, bioinformatic analyses including volcano plots, Venn plots, clustering analysis, Gene Ontology (GO), Kyoto Encyclopedia of Genes and Genomes (KEGG) pathway, gene sets enrichment analysis, and protein-protein interaction analysis were conducted.

**Results:**

In total, 1145 differentially methylated peaks, 2261 differentially expressed genes, and 671 differentially methylated and expressed genes (DMEGs) were identified. GO and KEGG pathway analyses conducted for these DMEGs revealed extensive and osteogenic biological functions. The “PI3K-Akt signaling pathway”; “MAPK signaling pathway”; “parathyroid hormone synthesis, secretion, and action”; and “p53 signaling pathway” were significantly enriched, and the DMEGs in these pathways were identified as m^6^A-specific key genes. A protein-protein interaction network based on DMEGs was built, and VEGFA, CD44, MMP2, HGF, and SPARC were speculated as the hub DMEGs.

**Conclusions:**

The total m^6^A level was reduced with osteogenic differentiation of hASCs. The transcriptome-wide m^6^A methylome built in the present study indicated quite a few signaling pathways, and hub genes were influenced by m^6^A modification. Future studies based on these epigenetic clues could promote understanding of the mechanisms of osteogenic differentiation of hASCs.

**Supplementary Information:**

The online version contains supplementary material available at 10.1186/s13287-021-02508-1.

## Introduction

Large bone defects, caused by trauma, bone loss, and tumors, lead to heavy social and economic burdens. At present, the clinical gold standard for the treatment of skeletal defects is autogenous or allogeneic bone grafts, which suffers disadvantages of a limited amount of harvested bone and donor site morbidity [1, 2]. Bone tissue engineering, built on stem cells or osteoprogenitor cells, osteoconductive biomaterials, and osteoconductive cytokines facilitating cellular proliferation and osteogenic differentiation, offers a promising solution [2, 3]. Human bone marrow-derived mesenchymal stem cells (hBMSCs), naturally resident in the bone marrow, are pluripotent and have been widely applied in bone tissue engineering for years [4, 5]. However, the access to autogenous hBMSCs is invasive and subject to limited cell incidence. What is more, the self-renewal and proliferative ability of hBMSCs weakens with donor aging and diseases such as osteoporosis and arthritis [6–8].

Adipose stromal cells, available from lipoaspirate, are a cellular niche containing differentiated cells, committed progenitors, and mesenchymal stem cells [9]. Among the multiple cell types, adipose-derived stem cells (ADSCs) are pluripotent, with adipogenic, chondrogenic, neurogenic, and osteogenic potentials. In the presence of ascorbate, glycerophosphate, and dexamethasone, ADSCs can differentiate into osteoblast-like cells [10–12]. Meanwhile, ADSCs have high proliferation rates and immunosuppressive properties and can secrete numerous polypeptides, hormones, and effective growth factors to induce osteogenesis. Moreover, ADSCs can also secrete a mass of suitable chemokines to recruit endogenous stem cells into the bone defect site [12–14]. With the increasing incidence of obesity worldwide, subcutaneous adipose tissue is easy to obtain, making human adipose-derived stem cells (hASCs) a first-rate alternative for hBMSCs in bone regenerative medicine [11].

Eukaryotic RNAs can be modified post-transcriptionally by over 170 modifications, among which, N^6^-methyladenosine (m^6^A), mostly resident at the stop codon and the 3′ untranslated region (UTR), is the most abundant [15, 16]. The m^6^A modification can be dynamically regulated by methyltransferases (also known as “writers”, including METTL3, METTL14, etc.) and demethylases (also known as “erasers,” including FTO, ALKBH5) and can further be recognized by specific RNA-binding proteins (also known as “readers,” including YTHDF1, YTHDF2) [17, 18]. As the first known form of reversible mRNA modification, m^6^A exerts effects on the stability, alternative splicing, export, cellular localization, and translation of RNAs, therefore influencing gene expression [17–20]. Till now, this epigenetic mark on RNAs has been extensively reported in virtually all major biological processes, normal development, and various diseases [21–26].

In the case of bone biology, m^6^A modification plays critical roles in bone development as well as bone diseases such as osteoporosis, osteoarthritis, and osteosarcoma [27, 28]. Mechanistically, m^6^A was found executing dual functions in the osteogenic differentiation of MSCs. On the one hand, m^6^A methylation enhanced osteogenic differentiation by regulating miR-320, RUNX2, Pth1r, PI3K/Akt, VEGF, or SMAD7/SMURF1 signaling, and inhibited adipogenic differentiation by regulating JAK1, Pth1r, SRSF2/ RUNX1T1, or TRAF4/ PKM2/β-catenin signaling [27–30]. On the other hand, the m^6^A methylation was also reported to decrease osteogenic differentiation by regulation of MYD88/NF-κB, miR-7212-5p/FGFR3, or FTO/p-AMPK loop [27, 28, 31]. However, all these findings above are rooted in BMSCs, and little is known about the scenario in ADSCs at the present stage.

In the present study, we aimed to investigate the roles of m^6^A in the osteogenic differentiation of hASCs. High-throughput sequencing for RNA (RNA-seq) and methylated RNA immunoprecipitation and sequencing (m^6^A MeRIP-Seq) were performed to comprehensively identify differentially regulated m^6^A peaks and messenger RNAs between two groups: undifferentiated hASCs (uhASCs) and osteogenically differentiated hASCs (dhASCs). The expressions of osteogenic markers, m^6^A regulating genes, and hub genes were validated by quantitative real-time polymerase chain reaction (qRT-PCR). Gene Ontology (GO) and Kyoto Encyclopedia of Genes and Genomes (KEGG) pathway analyses were performed for the differentially methylated genes (DMGs), differentially expressed genes (DEGs), and differentially methylated and expressed genes (DMEGs) to reveal the biological significance of m^6^A in osteogenic differentiation of hASCs. Moreover, we also built the protein-protein interaction (PPI) network based on the DMEGs and calculated the hub genes in this process. These findings could provide a new perspective on the molecular mechanisms of osteogenic differentiation in hASCs.

## Materials and methods

### Cell culture

The primary hASCs from healthy adult donors in this study were obtained from Cyagen Biosciences (Suzhou, China). The cells were cultured with OriCell™ Human ASC Growth Medium (Cyagen) containing 10% fetal bovine serum, 1% penicillin-streptomycin, and 1% glutamine. The medium was renewed every 3 days until the cells were passaged upon approximately 80% confluence. All the cells were cultured in an incubator with 5% CO_2_ at 37 °C. As our preliminary study indicated, the hASCs expressed a high level of stem cell surface markers CD29, CD44, CD105, and HLA-ABC, while less than 5% expressed CD31, CD34, CD45, and HLA-DR [32]. The third to the seventh generation of hASCs were used for osteogenic differentiation.

### Osteogenic differentiation

The hASCs were seeded at a density of 2.0 × 10^5^ and 1.0 × 10^5^ cells/well into six- and twelve-well plates, respectively. For osteogenic differentiation, hASCs were cultured in OriCell™ hASC Osteogenic Differentiation Medium (Cyagen) containing 10% fetal bovine serum, 1% penicillin-streptomycin, 10 mM β-glycerophosphate, 10 mM glutamine, 50 μM ascorbate, and 0.1 μM dexamethasone. For the control group, hASCs were cultured in the general growth medium as mentioned before. The medium was renewed every 2 days. Both groups were detected by alkaline phosphatase (ALP) and Alizarin Red S (ARS) staining, ALP activity assay, and qRT-PCR analysis of ALP, RUNX2, BGLAP, SPP1, SP7, and COL1A1 genes [33–35].

### ALP and ARS staining

ALP staining was conducted after 10 days of induction of osteogenic differentiation. Cells were fixed with 4% polyoxymethylene for 15 min and incubated with 0.1 M Tris buffer (pH 9.3) containing 0.25% naphthol AS-BI phosphate (Sigma) and 0.75% Fast Blue BB (Sigma) at 37 °C for 45 min. The Alkaline Phosphatase Assay Kit (Beyotime, China) was adopted according to the manufacturer’s instruction, and the ALP activity was quantified by a spectrophotometer (Thermo Fisher Scientific) at 405 nm.

ARS staining was conducted after 14 days of induction of osteogenic differentiation. Cells were fixed with 4% polyoxymethylene for 15 min and stained with 1% Alizarin Red S (ARS) Stain Solution (Cyagen) at room temperature for 5 min. And then, the solution was removed, and the samples were rinsed 3–4 times with deionized water. Alizarin Red-positive area was analyzed using the ImageJ software (NIH) and shown as a percentage of Alizarin Red-positive area over the total area.

### Quantitative real-time polymerase chain reaction analysis

The expressions of osteogenic markers, m^6^A regulating genes, and hub DMEGs were validated by qRT-PCR. Total RNA from hASCs was extracted using TRIzol Reagent (Invitrogen) according to the manufacturer’s instructions. The RNA purity and concentration were measured with a NanoDrop 2000 (Thermo Fisher Scientific). Reverse transcription was performed by PrimeScript™ RT Reagent Kit with gDNA Eraser (Takara, Japan). qRT-PCR reaction was performed using TB Green™ Premix Ex Taq™ II (Takara) in QuantStudio 3 Real-Time PCR Systems (Thermo Fisher Scientific). Relative gene expression was normalized by GAPDH using a 2−ΔΔCt method. The primers used in this study are listed in Supplementary Table S1.

### Western blot

Cells after 7 days of osteogenic induction were lysed in RIPA buffer (Beyotime, China) on ice. The samples were heated at 95 °C for 5 min in a sample buffer containing 20% SDS-PAGE sample loading buffer, separated on 10% SDS-polyacrylamide gels, and transferred to the PVDF membranes by a wet transfer apparatus (Bio-Rad). The membranes were blocked with 5% BSA for 1 h and then incubated overnight with rabbit METTL3 polyclonal antibody (1:1000, Cell Signaling Technology, Cat No: 96391), METTL14 polyclonal antibody (1:1000, ABclonal, Cat No: A8530), FTO polyclonal antibody (1:1000, Affinity, Cat No: DF8421), ALKBH5 polyclonal antibody (1:2000, Proteintech, Cat No: 16837-1-AP), and α-tubulin rabbit polyclonal antibody (1:2000, Beyotime, Cat No: AF0001), followed by incubation with a goat anti-rabbit IgG secondary antibody HRP conjugated (1:5000, Signaling antibody, Cat No: L3012-2). The antibody-antigen complexes were visualized with Bio-Rad Quantity One (Bio-Rad Laboratories Inc., Hercules, CA, USA).

### m^6^A level quantification

Total RNA from hASCs was extracted using TRIzol Reagent (Invitrogen) according to the manufacturer’s instructions. The RNA purity and concentration were measured with a NanoDrop 2000 (Thermo Fisher Scientific). The m^6^A RNA Methylation Quantification Kit (P-9005, EpiQuik) was used to measure the m^6^A content in the total RNAs following the manufacturer’s instruction. The m^6^A levels were quantified colorimetrically by reading the absorbance of each well at a wavelength of 450 nm, and the m^6^A level was then calculated based on the standard curve.

### m^6^A MeRIP-Seq and RNA-seq

The m^6^A MeRIP-Seq and RNA-Seq were performed after 7 days of induced osteogenic differentiation. Total RNA was extracted using TRIzol Reagent (Invitrogen, CA, USA) and was tested for quality and quantity with Bioanalyzer 2100 and RNA 6000 Nano LabChip Kit (Agilent, CA, USA) with RIN number > 7.0. Then, poly-T oligo-attached magnetic beads (Invitrogen) were used to isolate poly(A) mRNA from the total RNA. Next, the poly(A) mRNA fractions are fragmented into ~ 100-nt-long oligonucleotides and then incubated for 2 h at 4 °C with m^6^A-specific antibody (No. 202003, Synaptic Systems, Germany) in IP buffer (50 mM Tris-HCl, 750 mM NaCl and 0.5% Igepal CA-630) supplemented with BSA (0.5 μg μl^−1^). The mixture was then incubated with protein-A beads and eluted with elution buffer (1 × IP buffer and 6.7 mM m^6^A). Eluted RNA was precipitated by 75% ethanol. Both eluted m^6^A-containing fragments (IP) and untreated control fragments (input) are converted to the final cDNA library. The average insert size for the paired-end libraries was ~ 100 ± 50 bp. And then the paired-end 2 × 150 bp sequencing was performed on an Illumina Novaseq™ 6000 platform at the LC-BIO Bio-Tech Ltd. (Hangzhou, China) following the vendor’s recommended protocol.

### Data analysis

First, we used Cutadapt and perl scripts inhouse to remove the reads containing adaptor contamination, low-quality bases, and undetermined bases. Then, sequence quality was validated by fastp. Next, HISAT2 was used to map reads to the genome of *Homo sapiens* (version: v96). The R package exomePeak was adopted to process mapped reads of IP and input libraries and identifies m^6^A peaks with bed or bam format that can be adapted for visualization on the IGV software (http://www.igv.org/). HOMER and MEME were used for de novo and known motif finding followed by localization of the motif with respect to peak summit by perl. Called peaks were annotated by intersection with gene architecture using ChIPseeker. Then, StringTie was used to perform the expression level for all mRNAs from input libraries by calculating FPKM. The differentially expressed mRNAs were selected with the standard of *p*-value < 0.05 and log2 (fold change) > 1 or < −1 and by the R package edgeR.

### Venn analysis

Venn analysis was performed to characterize overlapped dysregulated m^6^A peaks and mRNAs between dhASCs and uhASCs detected by MeRIP-Seq and RNA-Seq. The number of m^6^A peaks and mRNAs with or without overlapping were shown in different colors in a pie diagram. Up- and downregulated m^6^A peaks and mRNAs were analyzed separately.

### GO and KEGG analyses

GO and KEGG pathway analyses were performed on m^6^A peaks and mRNAs. GO analysis, including molecular function, biologic process, and cellular component, was performed. *p* < 0.05 denoted the significance of GO term enrichment. Based on the KEGG database, pathway analysis was performed to analyze the potential functions. GO and pathway enrichment analyses were also performed based on the differentially regulated m^6^A peaks and mRNAs.

### Gene set enrichment analysis

For gene set enrichment analysis (GSEA), gene lists “c2.cp.kegg.v7.3.symbols” were obtained from MSigDB, and the analysis was performed with the GSEA software (http://www.broad.mit.edu/GSEA). In short, we imported our gene lists of interest into the software and examined the significance of the given gene sets. *p*-values were computed using a bootstrap distribution created by resampling gene sets of the same cardinality.

### Construction of the protein-protein interaction network

The protein-protein interaction (PPI) network was constructed to assess the interactions between the differentially methylated and expressed genes (DMEGs) based on the correlation analysis. All the DMEGs were selected to construct the network using Cytoscape 3.8 (Institute of Systems Biology in Seattle). Then, we calculated the hub genes by combining scores from all DMEGs.

### Statistical analysis

Statistical tests were performed using R version 3.6.0, SPSS 24.0 (IBM, NY, USA) and GraphPad Prism 8.0. Numerical data was presented as means ± standard deviation. Differences between the two groups were evaluated for statistical significance by a *t*-test. The appropriate analysis of variance was used when more than two groups were evaluated, followed by Dunnett’s multiple comparisons test to compare the difference between the groups. *p* ≤ .05 was considered statistically significant. The R packages VennDiagram, ComplexHeatmap, clusterProfiler, and ggplot2 were used for graphing and data visualization.

## Results

### Osteogenic differentiation of hASCs was successfully induced.

**Fig. 1:**
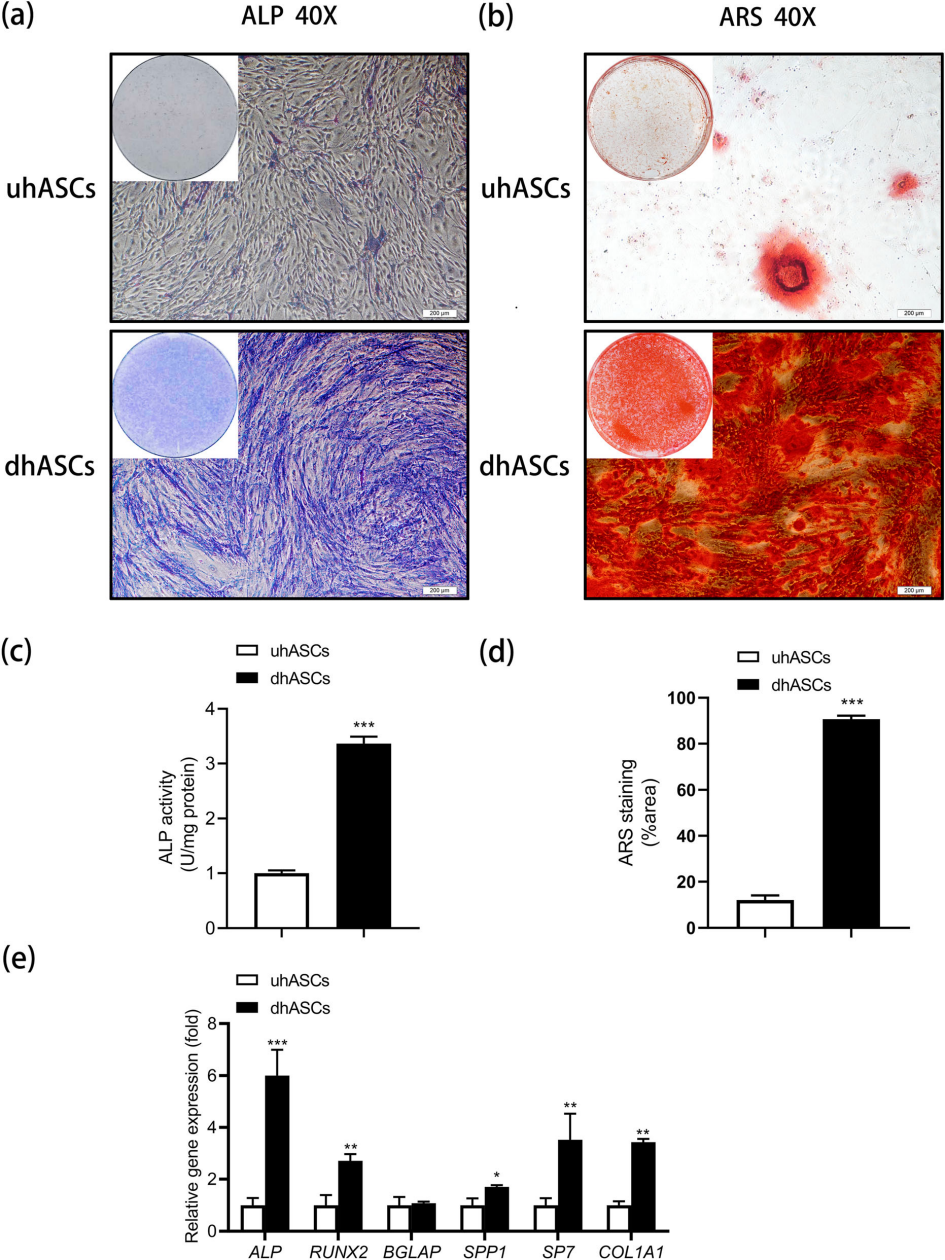
Osteogenic differentiation of hASCs was successfully induced. **a** Representative images of ALP staining. **b** Representative images of ARS staining. **c** Quantitative analyses of ALP activity. **d** Quantitative analyses of ARS staining. **e** qRT-PCR analyses of the expression of ALP, RUNX2, BGLAP, SPP1, SP7, and COL1A1 genes. *p < .05, **p < .01, ***p < .001. ALP, alkaline phosphatase; ARS, Alizarin Red S; hASCs, human adipose-derived stem cells; qRT-PCR, quantitative real-time polymerase chain reaction; uhASCs, undifferentiated hASCs; dhASCs, osteogenically differentiated hASCs

The ALP activity, validated by ALP staining and ALP activity quantification assay, was significantly higher in the dhASCs group than in the uhASCs group. A similar result regarding mineral nodes forming was observed in ARS staining (Fig. 1a–d). Moreover, compared to the control group, the expressions of ALP, RUNX2, SPP1, SP7, and COL1A1 were significantly increased in the dhASCs group (Fig. 1e). These results confirmed the osteogenic differentiation of dhASCs.

### m^6^A level significantly decreased during osteogenic differentiation of hASCs

**Fig. 2:**
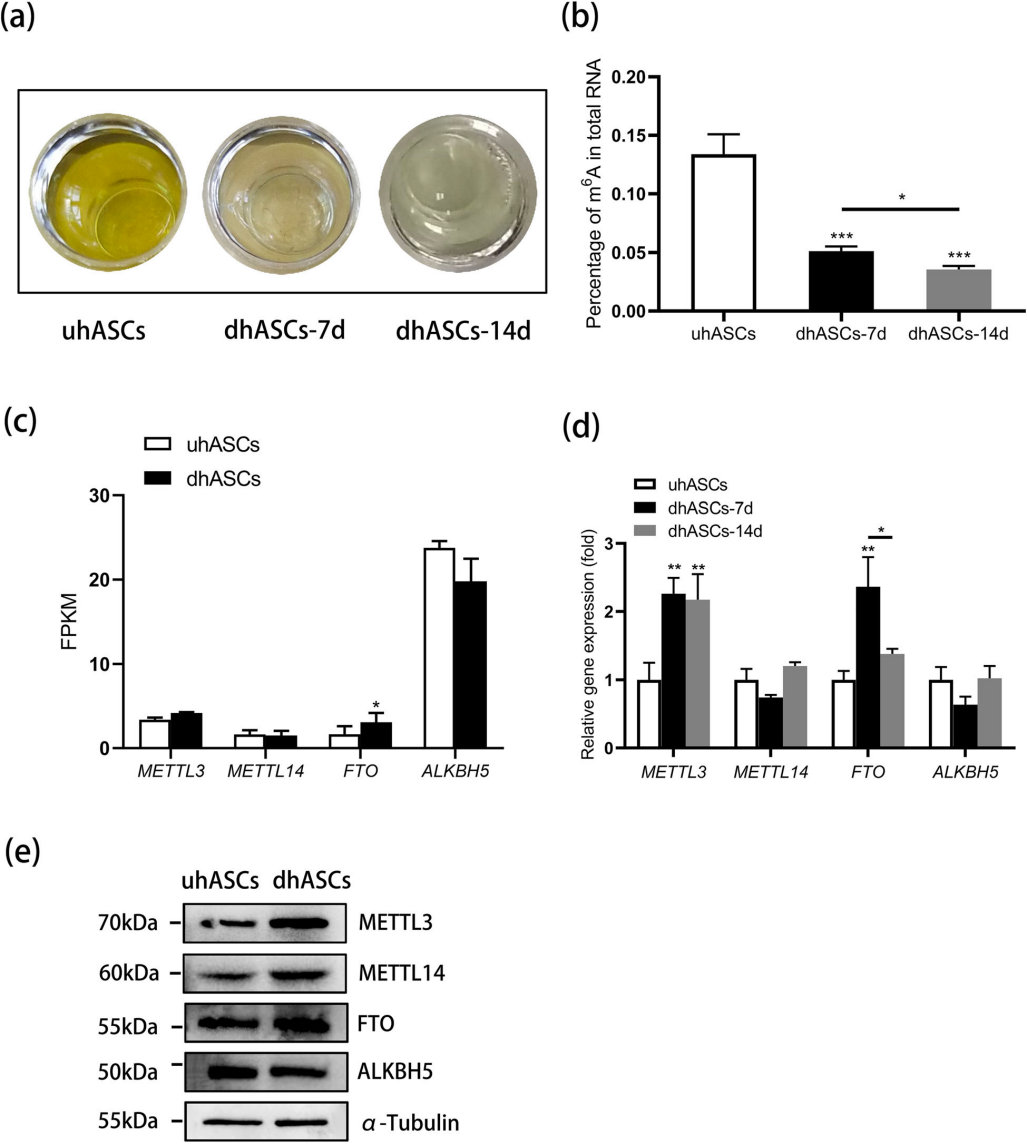
m^6^A level significantly decreased during osteogenic differentiation of hASCs. **a** Representative image of m^6^A level quantification assay. **b** Quantitative analyses of m^6^A level. **c** Expression of m^6^A regulators during the osteogenic differentiation of hASCs by RNA-seq (uhASCs vs dhASCs-7 day). **d** qRT-PCR analyses of the expression of *METTL3*, *METTL14*, *FTO*, and *ALKBH5*. **e** Western blot analysis of METTL3, METTL14, FTO, and ALKBH5 (uhASCs vs dhASCs-7 day). *p < .05, **p < .01, ***p < .001. hASCs, human adipose-derived stem cells; qRT-PCR, quantitative real-time polymerase chain reaction; uhASCs, undifferentiated hASCs; dhASCs, osteogenically differentiated hASCs

To learn about the m^6^A methylation in the process of osteogenic differentiation of hASCs, we first detected the relative m^6^A level between the groups. The m^6^A quantification assay analysis revealed a significantly reduced m^6^A level during the osteogenic induction of hASCs from day 0 to day 14, with the most significant and steepest reduction at the early stage (Fig. 2a, b). Then, we investigated the expression of m^6^A regulators. According to the RNA-seq, compared with the uhASCs group, FTO expression was significantly increased in the dhASCs group at day 7. However, the expression of METTL3, METTL14, and ALKBH5 showed no significant differences between the groups (Fig. 2c). Further, the qRT-PCR revealed the expression of METTL3 was significantly elevated at day 7 and day 14, and the expression of FTO was significantly elevated at day 7 and significantly decreased to baseline at day 14 (Fig. 2d). Moreover, the expressions of METTL3, METTL14, FTO, and ALKBH5 between the groups were confirmed by western blotting (Fig. 2e). In a word, these results demonstrated potential effects of m^6^A in the osteogenic differentiation of hASCs.

### m^6^A peaks were significantly dysregulated during osteogenesis of hASCs

**Fig. 3:**
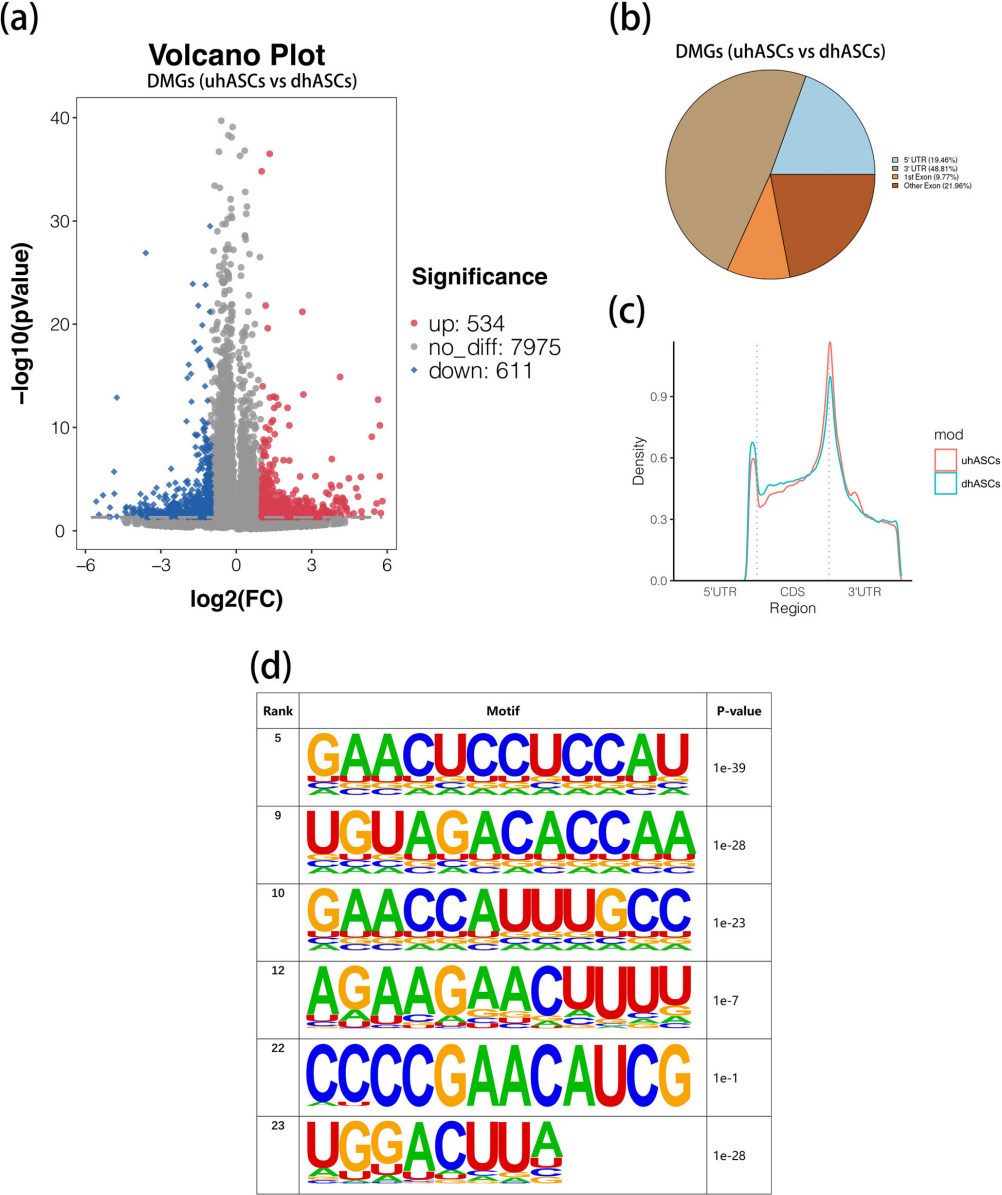
m^6^A peaks were significantly dysregulated during osteogenesis of hASCs. **a** Volcano plot displaying the DMGs (fold change ≥ 2 and *p* < 0.05). **b** Pie chart showing distribution of differentially methylated m^6^A peaks in different gene contexts. **c** Accumulation of differentially methylated m^6^A peaks along transcripts. **d** The m^6^A motifs significantly enriched across m^6^A peaks identified between uhASCs and dhASCs. DMGs, differentially methylated genes; hASCs, human adipose-derived stem cells; uhASCs, undifferentiated hASCs; dhASCs, osteogenically differentiated hASCs; FC, fold change

To dissect the underlying roles of m^6^A in the osteogenesis of hASCs, we performed the m^6^A MeRIP-Seq to depict the critical picture of m^6^A methylome. For the whole transcriptome, 39,735 (20431 transcripts) and 44,630 (22789 transcripts) m^6^A peaks were detected in uhASCs and dhASCs, respectively. For uhASCs, 45.73% of the peaks were detected in the 3′UTR region, 18.39% in the 5′UTR region, 10.6% in the first exon, and 25.28% in the remaining exons (Supplementary Figure S1a-b). For dhASCs, 43.85% of the peaks were detected in the 3′UTR region, 20.48% in the 5′UTR region, 11.06% in the first exon, and 24.61% in the remaining exons (Supplementary Figure S1c-d). According to the MeRIP-Seq, by the standard of *p*-value < 0.05 and fold change (fc) ≥ 2, 1145 m^6^A peaks were significantly dysregulated after a 7-day osteogenic induction of hASCs, of which 534 peaks were hypermethylated and 611 hypomethylated (Fig. 3a). In addition, 48.81% of the dysregulated peaks were in the 3′UTR region, 19.46% in the 5′UTR region, 9.77% in the first exon, and 21.96% in the remaining exons (Fig. 3b, c). The top 20 DMGs were listed in Table 1, and the full list of DMGs was shown in Supplementary Table S2. Furthermore, the motif analysis validated m^6^A RRACH (R was purine, A was m^6^A, and H was a non-guanine bas) consensus sequence enrichment between uhASCs and dhASCs (Fig. 3d).

**Table 1:** The top 20 differently methylated m6A peaks (uhASCs/dhASCs)

Gene	Transcript ID	Chr	Start	End	*p*-value	FC	Regulation
ASCL2	ENST00000331289	chr11	2268644	2268853	0.00	56.10	Hyper
DTX2P1-UPK3BP1-PMS2P11	ENST00000636308	chr7	77024167	77024406	0.02	53.82	Hyper
OR2A9P	ENST00000484189	chr7	144299355	144299534	0.00	52.35	Hyper
SLX1B	ENST00000330181	chr16	29457634	29457951	0.00	52.35	Hyper
SLX1B	ENST00000330181	chr16	29457636	29457873	0.00	49.87	Hyper
C4A	ENST00000428956	chr6	31983915	31984330	0.00	47.84	Hyper
FP565260	ENST00000623998	chr21	5158279	5158428	0.01	44.94	Hyper
MAGED4	ENST00000599522	chrX	52187058	52187386	0.00	41.93	Hyper
AC092296	ENST00000589470	chr19	36421976	36422123	0.05	32.00	Hyper
AC002066	ENST00000446355	chr7	116276826	116277005	0.01	31.56	Hyper
SERF1A	ENST00000317633	chr5	70901805	70905325	0.00	31.12	Hyper
NUDT4B	ENST00000322209	chr1	148748953	148749247	0.00	0.02	Hypo
CHST8	ENST00000650847	chr19	33772791	33773000	0.00	0.02	Hypo
C11orf44	ENST00000317019	chr11	130715177	130715475	0.03	0.02	Hypo
MYCL	ENST00000397332	chr1	39895965	39896534	0.00	0.03	Hypo
GOSR2	ENST00000640792	chr17	46964434	46964733	0.04	0.03	Hypo
ATF3	ENST00000366981	chr1	212618623	212618743	0.01	0.03	Hypo
AADAC	ENST00000232892	chr3	151814103	151814251	0.05	0.03	Hypo
GOLGA8N	ENST00000448387	chr15	32599455	32599770	0.00	0.03	Hypo
PSMC1	ENST00000553835	chr14	90267920	90268160	0.00	0.03	Hypo
BSN	ENST00000296452	chr3	49655753	49655873	0.04	0.04	Hypo

*dhASCs*, osteogenically differentiated human adipose-derived stem cells; *uhASCs*, undifferentiated human adipose-derived stem cells; *FC*, fold change

### The DMGs were associated with extensive and osteogenic biological functions

**Fig. 4:**
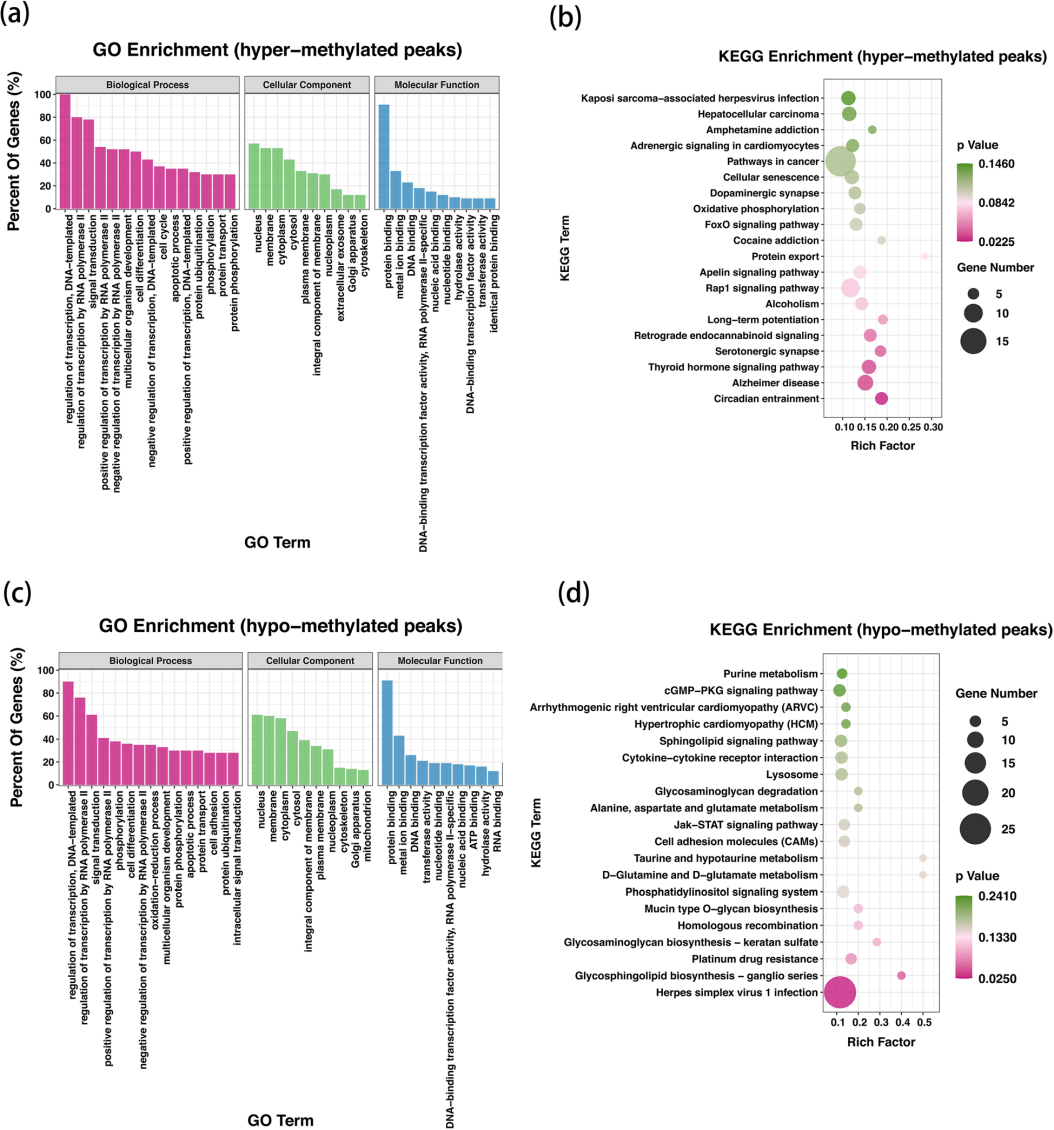
The DMGs were associated with extensive and osteogenic biological functions. **a** GO enrichment analysis for hypermethylated peaks. **b** KEGG pathway analysis for hypermethylated peaks. **c** GO enrichment analysis for hypomethylated peaks. **d** KEGG pathway analysis for hypomethylated peaks. GO, Gene Ontology; KEGG, Kyoto Encyclopedia of Genes and Genomes

To figure out the biological significance of m^6^A methylation in the osteogenesis of hASCs, we performed GO and KEGG pathway analyses for DMGs. GO analysis uncovered that both the hypermethylated and hypomethylated genes in dhASCs were significantly involved with “regulation of transcription, DNA-templated,” “regulation of transcription by RNA polymerase II,” “signal transduction,” “cell differentiation,” and “multicellular organism development” (ontology: biological process); “membrane,” “nucleus,” and “cytoplasm” (ontology: cellular component); and “protein binding,” “metal ion binding,” and “DNA binding” (ontology: molecular function) (Fig. 4a, c). Remarkably, KEGG pathway analysis demonstrated that the hypermethylated genes were significantly associated with signaling pathway “thyroid hormone signaling pathway,” “Rap1 signaling pathway,” and “FoxO signaling pathway” (Fig. 4b). However, the hypomethylated genes were not significantly associated with the common osteogenic signaling pathway (Fig. 4d).

### Osteogenic pathways and genes were significantly differentially methylated and regulated

**Fig. 5:**
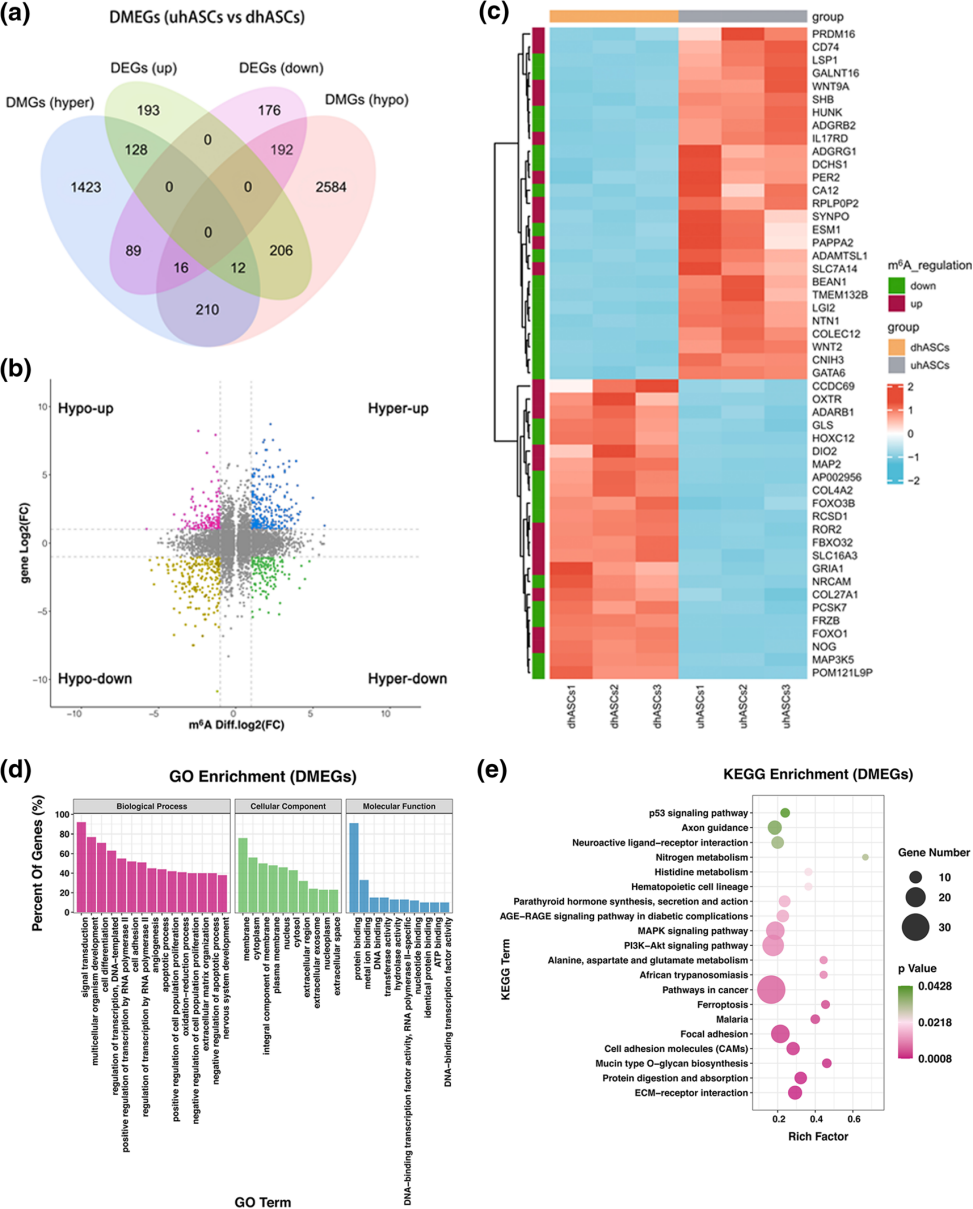

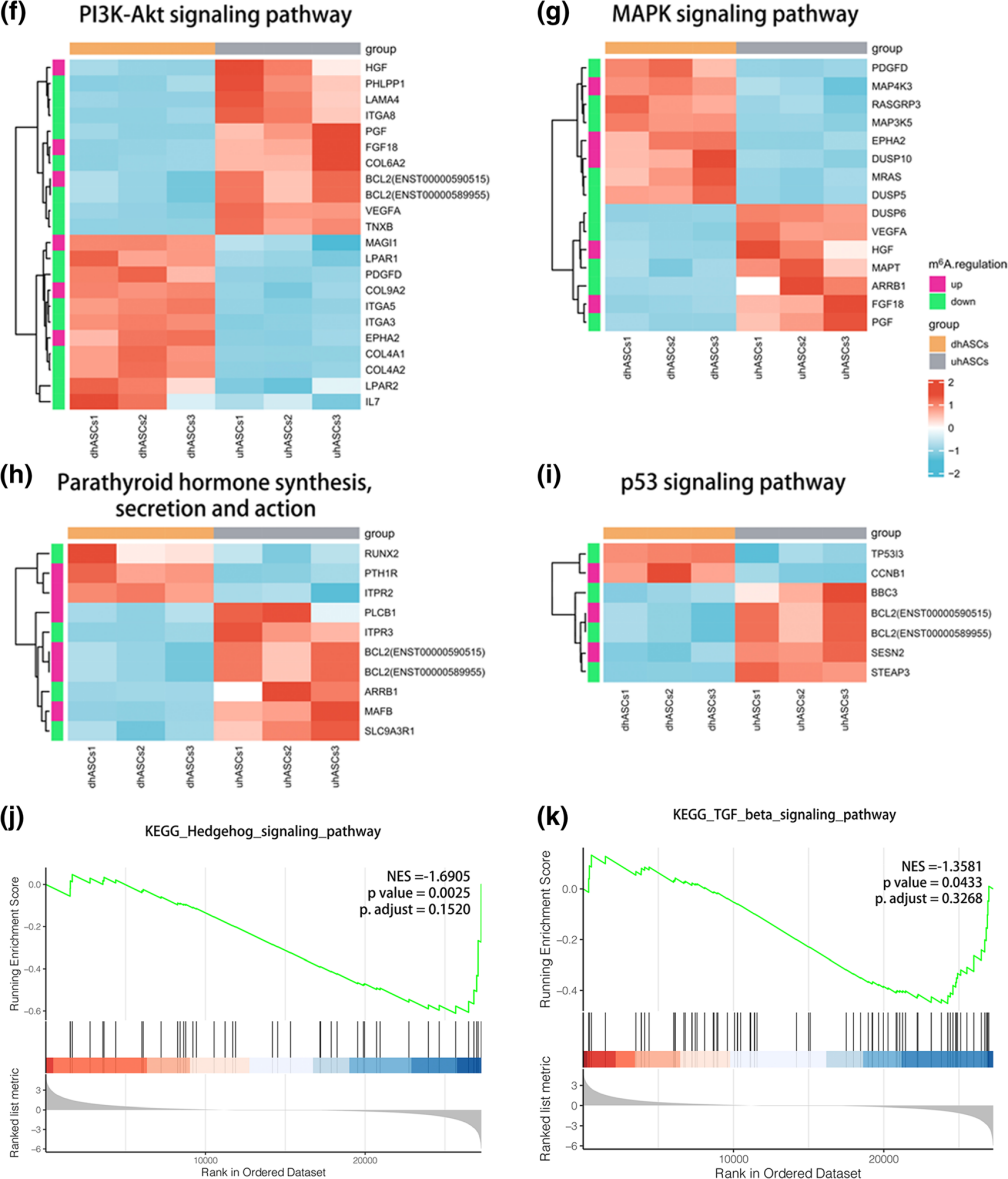
Osteogenic pathways and genes were significantly differentially methylated and regulated. **a** Venn plot for DMEGs. **b** Four-quadrant graph for hyper-up, hypo-down, hyper-down, and hypo-up DMEGs. **c** Hierarchical clustering analysis of the top 50 DMEGs (by the standard fold change ≥ 2 and *p* < 0.05 for both DEGs and DMGs, ranking by *p*-value of DEGs). **d** GO enrichment analysis for DMEGs. **e** KEGG pathway analysis for DMEGs. **f** Hierarchical clustering analysis of DMEGs in the PI3K-Akt signaling pathway. **g** Hierarchical clustering analysis of DMEGs in the MAPK signaling pathway. **h** Hierarchical clustering analysis of DMEGs in the pathway of parathyroid hormone synthesis, secretion, and action. **i** Hierarchical clustering analysis of DMEGs in the P53 signaling pathway. **j** GSEA showed decreased enrichment of the Hedgehog signaling pathway. **k** GSEA showed decreased enrichment of the TGF-beta signaling pathway. DEGs, differentially expressed genes; DMGs, differentially methylated genes; DMEGs, differentially methylated and expressed genes; hASCs, human adipose-derived stem cells; uhASCs, undifferentiated hASCs; dhASCs, osteogenically differentiated hASCs; hyper-up, hypermethylated and upregulated genes; hypo-down, hypomethylated and downregulated genes; hyper-down, hypermethylated and downregulated genes; hypo-up, hypomethylated and upregulated genes; GO, Gene Ontology; KEGG, Kyoto Encyclopedia of Genes and Genomes; GSEA, gene set enrichment analysis

To figure out the alteration of gene expression and signaling pathways in the osteogenesis of hASCs, we performed RNA-seq to figure out the DEGs. By the standard of *p*-value < 0.05 and fc ≥ 2, compared with uhASCs, 2261 genes were significantly dysregulated in the dhASCs group, of which 1210 were upregulated and 1051 were downregulated (Supplementary Figure S2a). The hierarchical clustering of the top 50 DEGs was shown in Supplementary Figure S2b and the full list of DEGs was shown in Supplementary Table S3. GO analysis and KEGG pathway analysis of DEGs were shown in Supplementary Figure S2b-e. By conjoint analysis of DMGs (by the standard of *p*-value < 0.05) and DEGs (by the standard of *p*-value < 0.05 and fold change (fc) ≥ 2), we found 671 differentially methylated and expressed transcripts (598 genes) were mainly divided into four groups, including 140 hypermethylated and upregulated (hyper-up), 208 hypomethylated and downregulated (hypo-down), 105 hypermethylated but downregulated (hyper-down), and 218 hypomethylated but upregulated genes or transcripts (hypo-up) (Fig. 5a, b). The hierarchical clustering of the top 50 DMEGs was shown in Fig. 5c. The top 20 DMEGs were listed in Table 2, and the full list of DMEGs was shown in Supplementary Table S4. GO analysis uncovered that the DMEGs in dhASCs were significantly involved with “signal transduction,” “multicellular organism development,” “regulation of transcription, DNA-templated,” “cell differentiation,” “cell adhesion,” and “regulation of cell population proliferation” (ontology: biological process); “membrane,” “cytoplasm,” and “integral component of membrane” (ontology: cellular component); and “protein binding,” “metal ion binding,” and “DNA binding” (ontology: molecular function) (Fig. 5d). Remarkably, the KEGG pathway analysis demonstrated that the DMEGs were significantly associated with signaling pathway “PI3K-Akt signaling pathway,” “MAPK signaling pathway,” “parathyroid hormone synthesis, secretion and action,” and “p53 signaling pathway” (Fig. 5e). The DMEGs associated with these four signaling pathways were selected and performed a hierarchical cluster analysis (Fig. 5f–i). Furthermore, GSEA analysis of the whole transcriptome revealed these genes were significantly associated with the “Hedgehog signaling pathway” and the “TGF-beta signaling pathway” (Fig. 5j, k).

**Table 2:** The top 20 differently expressed genes containing differently methylated peaks (uhASCs/dhASCs)

Gene	Transcript ID	Chr	Start	End	m6A regulation	FC	*p*-value	Gene regulation
COLEC12	ENST00000400256	chr18	318935	319136	hypo	0.04	0.00	Down
FOXO1	ENST00000636651	chr13	40556102	40556341	hyper	42.51	0.00	Up
LSP1	ENST00000311604	chr11	1890072	1890311	hypo	0.07	0.00	Down
NRCAM	ENST00000522550	chr7	108149746	108156434	hypo	37.52	0.00	Up
FBXO32	ENST00000517956	chr8	123541116	123541206	hyper	9.41	0.00	Up
POM121L9P	ENST00000414583	chr22	24252274	24252423	hypo	8.26	0.00	Up
SLC7A14	ENST00000231706	chr3	170463356	170463596	hyper	0.05	0.00	Down
NOG	ENST00000332822	chr17	56594172	56594704	hyper	187.37	0.00	Up
CNIH3	ENST00000272133	chr1	224740106	224740315	hypo	0.04	0.00	Down
FRZB	ENST00000295113	chr2	182834653	182834922	hypo	296.94	0.00	Up
TMEM132B	ENST00000299308	chr12	125657702	125658152	hypo	0.10	0.00	Down
MAP2	ENST00000475600	chr2	209696191	209696621	hyper	28.70	0.00	Up
SHB	ENST00000377707	chr9	37916645	37916914	hyper	0.16	0.00	Down
GALNT16	ENST00000337827	chr14	69352155	69352903	hypo	0.09	0.00	Down
AP002956	ENST00000264036	chr11	119308798	119309008	hypo	12.46	0.00	Up
RCSD1	ENST00000537350	chr1	167697240	167705078	hypo	242.98	0.00	Up
DIO2	ENST00000422005	chr14	80200075	80200314	hyper	55.65	0.00	Up
OXTR	ENST00000316793	chr3	8750557	8750826	hyper	11.17	0.00	Up
ADARB1	ENST00000460734	chr21	45145486	45146205	hyper	7.92	0.00	Up
CD74	ENST00000009530	chr5	150400041	150400190	hyper	0.13	0.00	Down

*dhASCs*, osteogenically differentiated human adipose-derived stem cells; *uhASCs*, undifferentiated human adipose-derived stem cells; *FC*, fold change

### PPI network and hub genes were discovered among DMEGs

**Fig. 6:**
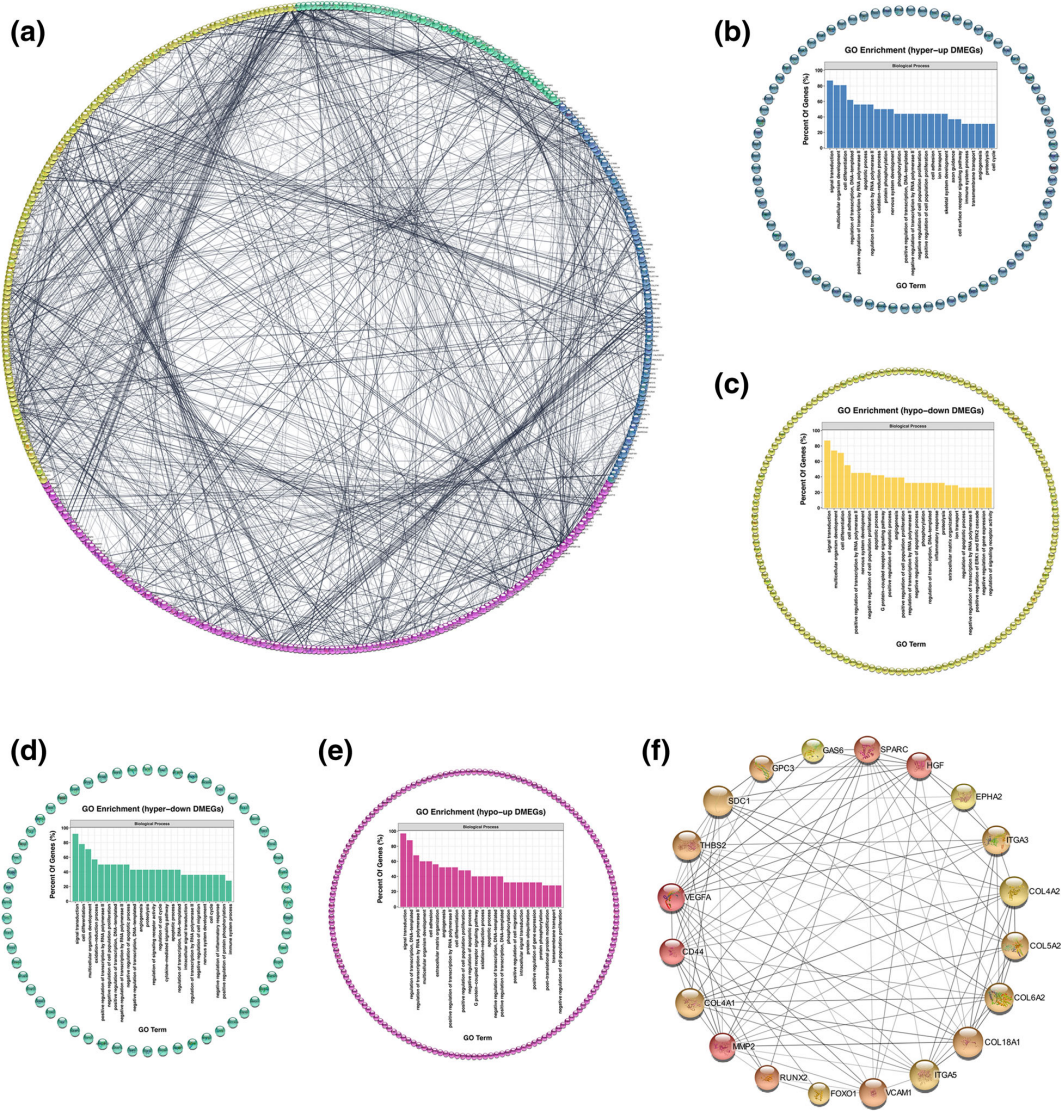

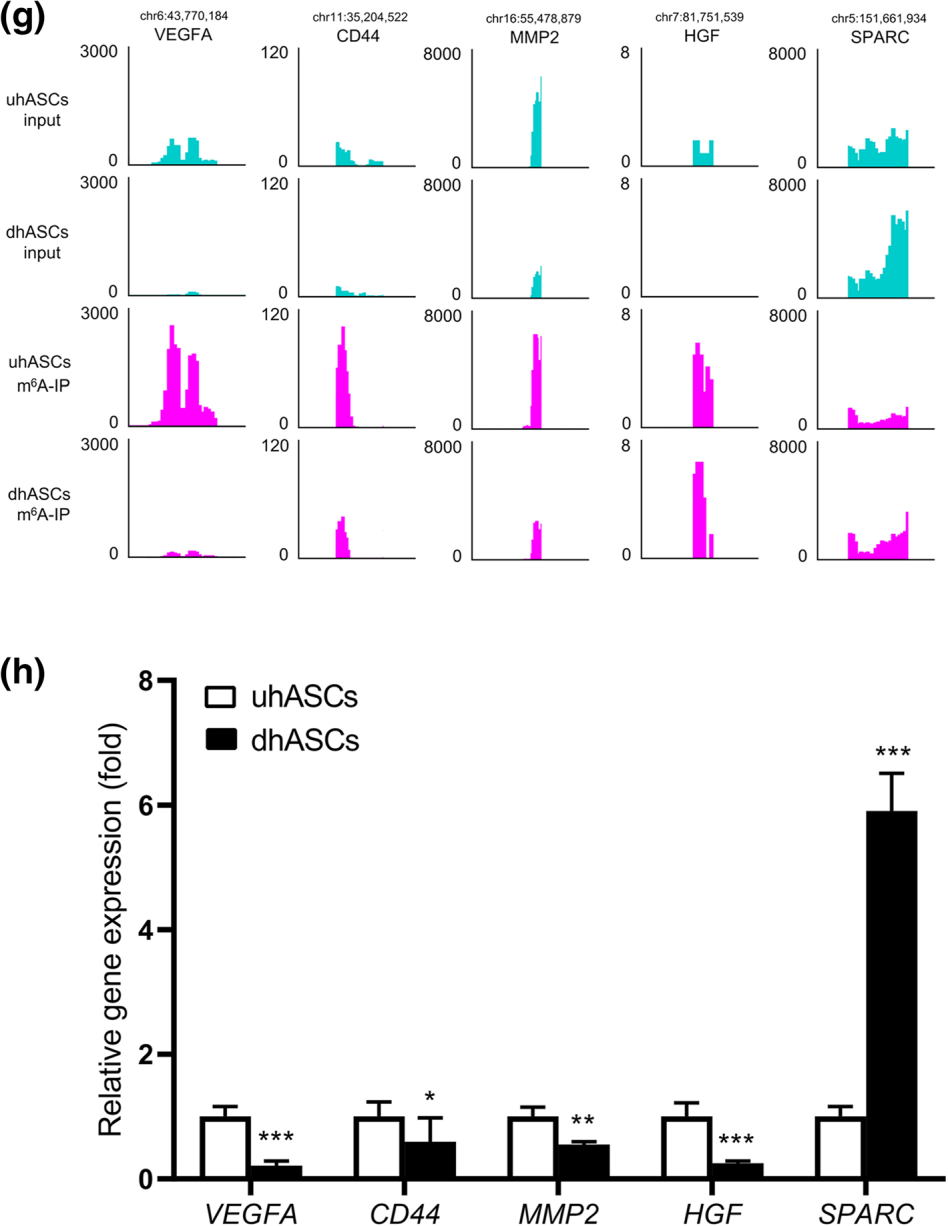
PPI network and hub genes were discovered among DMEGs. **a** PPI network of DMEGs. Blue represents the hypermethylated and upregulated genes, yellow represents the hypomethylated and downregulated genes, green represents the hypermethylated but downregulated genes, and red represents the hypomethylated but upregulated genes. **b** The hyper-up cluster and the GO enrichment analysis for DMEGs in this cluster. **c** The hypo-down cluster and the GO enrichment analysis for DMEGs in this cluster. **d** The hyper-down cluster and the GO enrichment analysis for DMEGs in this cluster. **e** The hypo-up cluster and the GO enrichment analysis for DMEGs in this cluster. **f** Hub genes calculated from the PPI network of DMEGs. The size and color of the nodes are proportional to the combining score. **g** Data visualization of VEGFA, CD44, MMP2, HGF, and SPARC mRNA m^6^A modification in uhASCs and dhASCs. **h** qRT-PCR analyses of the expression of hub DMEGs of VEGFA, CD44, MMP2, HGF, and SPARC. *p < .05, **p < .01, ***p < .001. PPI, protein-protein interaction; GO, Gene Ontology; KEGG, Kyoto Encyclopedia of Genes and Genomes; DMEGs, differentially methylated and expressed genes; hyper-up, hypermethylated and upregulated genes; hypo-down, hypomethylated and downregulated genes; hyper-down, hypermethylated and downregulated genes; hypo-up, hypomethylated and upregulated genes; hASCs, human adipose-derived stem cells; uhASCs, undifferentiated hASCs; dhASCs, osteogenically differentiated hASCs; qRT-PCR, quantitative real-time polymerase chain reaction

The PPI network of the DMEGs was conducted by STRING database and Cytoscape (Fig. 6a). The network was grouped into four clusters as mentioned before, that is, hyper-up, hypo-down, hyper-down, and hypo-up DMEGs (Fig. 6b–e). GO enrichment analyses were performed for each cluster of DMEGs to elucidate their biological function (Fig. 6b–e). Hub genes in the PPI network were selected using cytoHubba by calculating the protein combining degree (Fig. 6f). And the m^6^A peak distribution of the top 5 hub genes between the groups was shown in Fig. 6g, and the expressions of the 5 hub DMEGs were shown in Fig. 6h. The PPI network interaction metadata was listed in Supplementary Table S5.

## Discussion

Adipose-derived stem cells (ADSCs), as one form of mesenchymal stem cells with good accessibility and special biological properties, are frequently used for bone regeneration both in vitro and in vivo [11, 12]. According to the current criteria, the isolation of ADSCs produces cell type heterogeneity, and the purity of ADSCs is essential for their engineering use [9]. In the present study, the hASCs were identified with surface markers and successfully induced into osteoblast-like cells [9, 32]. Identifying factors involved in the osteogenic differentiation of ASCs is important for better understanding the mechanism of osteogenic differentiation. Recent studies have shown that m^6^A modification plays essential roles in stem cell fate decisions in a stage-, state-, and cell type-specific manner [26, 36–40]. In the case of osteogenic differentiation, reduced m^6^A level in BMSCs inhibited osteogenic differentiation by affecting the PTH/Pth1r signaling axis, the PI3K-AKT signaling pathways, or the alternative splicing of VEGFA [30, 40, 41]. As for preadipocytes, some studies reported FTO promoted adipogenesis by regulating cell cycle-related genes and the JAK2-STAT3-C/EBPb signaling pathway in an m^6^A-dependent manner in 3T3-L1 cell lines [42–45]. However, at the present stage, little is known about the roles of m^6^A modification in the osteogenic differentiation of ADSCs. In this study, using m^6^A MeRIP-Seq and RNA-seq, we depicted the transcriptome-wide m^6^A methylome in the process of osteogenic differentiation of hASCs for the first time.

In the present study, we observed that from day 0 to day 14, the level of m^6^A modification decreased continuously with the most significant and steepest reduction at the early stage. Therefore, we investigated the time point day 7 to explore potential m^6^A-related signaling pathways. Combined with significant expression changes of m^6^A regulators METTL3 and FTO, we unveiled the potential effects of m^6^A modification in osteogenic differentiation of hASCs. The changes of m^6^A level during osteogenic induction of hASCs appeared stage-specific. This was probably because of the time-dependent expression pattern of m^6^A regulators. At the early stage, the elevated expression of demethylase FTO predominantly reduced the m^6^A level. With the osteogenic induction lasting, the expression of FTO decreased while the expression of methyltransferase METTL3 maintained, resulting in a slower reduction of m^6^A level. Interestingly, this trend was contrary to that of BMSCs where a time-dependent increase of m^6^A content during osteogenic differentiation was reported [29]. In the cases of BMSCs, from day 0 to day 14, the expression of methyltransferase METTL3 increased while the expression of demethylase FTO and ALKBH5 did not change significantly [41]. The differences between hASCs and BMSCs might reflect the cell type-specific effects of m^6^A modification in stem cell differentiation [26, 40]. Although both were mesenchymal stem cells, the distinct origins may cause different gene expression pattern and epigenetic modifications, thus producing divergent stemness, proliferation capacity, multilineage differentiation potential, longevity, immunoregulatory properties, and so on [6, 46]. Considering the complex stage- and state-specific roles of m^6^A modification, the m^6^A methylome of a prolonged osteogenic induction of hASCs remains to be revealed.

In total, the m^6^A MeRIP-Seq revealed an abundance of 1.94 and 1.96 peaks per transcript for uhASCs and dhASCs, respectively. Additionally, the m^6^A peaks of both uhASCs and dhASCs were mostly enriched in the 3′UTR region and near the stop codon; these sites were m^6^A-specific and consistent with previous studies [15–19, 47]. Furthermore, during the osteogenic differentiation of hASCs, the differentially methylated peaks were significantly enriched in “signal transduction,” “regulation of transcription,” “multicellular organism development,” “cell differentiation,” and so on, indicating the conservative and fundamental roles of m^6^A in regulating development and cell fate specification [16, 17].

In order to elucidate the mechanism of m^6^A in affecting osteogenic differentiation of hASCs, we combined the m^6^A methylome and the transcriptome and found the key signaling pathways that were affected by m^6^A modification. As with previous studies, several canonical pathways regarding osteogenic differentiation were significantly enriched. The most significant ones were the “PI3K-Akt signaling pathways,” “MAPK signaling pathways,” “parathyroid hormone synthesis, secretion and action,” and “P53 signaling pathways,” in which quite a few key genes were differentially methylated [20, 22, 30, 40, 41, 48]. Moreover, although other common osteogenic pathways were not significantly enriched in the m^6^A methylome, quite a few key genes in these pathways were significantly methylated. At the same time, we constructed the PPI network of DMEGs and speculated the hub genes based on combining degree, for example, the genes of VEGFA, CD44, MMP2, HGF, and SPARC. All these differentially expressed as well as differentially methylated key genes and hub genes provided the latest and detailed information about how m^6^A modification affected the osteogenic differentiation of hASCs.

A mass of studies in the past few years have lighted up the biological effects of m^6^A modification on RNAs [19–22, 40, 47, 49–52]. On the one hand, the m^6^A methylation process is reversable, and this mark on RNA could be written or erased in case of various stimuli and biological factors [17, 47, 49]. On the other hand, m^6^A can affect RNA processing and metabolism in multifaceted mechanisms, including alternative splicing, alternative polyadenylation, RNA stability, RNA export, RNA degradation, and translation. Therefore, m^6^A up- or downregulates the gene expression in a complex and context-depended manner [17–19]. For this reason, we observed four groups of DMEGs in the present study, those were hyper-up, hypo-down, hyper-down, and hypo-up DMEGs. Our function enrichment analyses suggested that these four clusters of DMEGs were associated with the fundamental and different biological processes. Combined with the methylation and expression pattern of the key DMEGs, future studies based on these molecular clues could give us brand-new knowledge on the osteogenic differentiation of hASCs from an m^6^A-specific epigenetic perspective.

## Conclusions

Total m^6^A level was reduced with osteogenic differentiation of hASCs, and the transcriptome-wide m^6^A methylome built in the present study indicated quite a few signaling pathways and hub genes were influenced by m^6^A modification. In total, 1145 DMGs, 2261 DEGs, and 671 DMEGs were detected. GO and KEGG pathway analysis conducted for these differentially methylated and regulated genes revealed extensive and osteogenic biological functions. The “PI3K-Akt signaling pathway,” “MAPK signaling pathway,” “parathyroid hormone synthesis, secretion and action,” and “p53 signaling pathway” were significantly enriched, and the DMEGs in these pathways were identified as m^6^A-specific key genes. PPI network based on DMEGs was built, and VEGFA, CD44, MMP2, HGF, and SPARC were speculated as the hub DMEGs. Future studies based on these epigenetic clues could promote the understanding of the mechanisms of osteogenic differentiation of hASCs.

## Supplementary Information

**Additional file 1: Figure S1.** m6A peak calling for uhASCs and dhASCs. a Pie charts showing distribution of m^6^A peaks of uhASCs in different gene context. b Accumulation of m^6^A peaks of uhASCs along transcripts. c Pie charts showing distribution of m^6^A peaks of dhASCs in different gene context. d Accumulation of m^6^A peaks of dhASCs along transcripts. hASCs, human adipose-derived stem cells; uhASCs, undifferentiated hASCs; dhASCs, osteogenically differentiated hASCs.

**Additional file 2: Figure S2.** Gene expression profile during osteogenesis of hASCs by RNA-seq. a Volcano plots displaying the DEGs (fold change ≥ 2 and *p* < 0.05). b Hierarchical clustering analysis of the top 50 DEGs (ranking by *p* value). c GO enrichment analysis for upregulated genes. d KEGG pathway analysis for upregulated genes. e GO enrichment analysis for downregulated genes. f KEGG pathway analysis for downregulated genes. DEGs, differentially expressed genes; hASCs, human adipose-derived stem cells; uhASCs, undifferentiated hASCs; dhASCs, osteogenically differentiated hASCs; GO, gene ontology; KEGG, Kyoto Encyclopedia of Genes and Genomes; FC, fold change.

**Additional file 3: Table S1.** Primers used for qRT-PCR.

**Additional file 4: Table S2.** Full list of DMGs (uhASCs vs dhASCs).

**Additional file 5: Table S3.** Full list of DEGs (uhASCs vs dhASCs).

**Additional file 6: Table S4.** Full list of DMEG (uhASCs vs dhASCs).

**Additional file 7: Table S5.** The PPI network metadata.

## Data Availability

All sequencing data have been deposited into the Sequence Read Archive (SRA) database of NCBI with the identifier PRJNA739515 (https://dataview.ncbi.nlm.nih.gov/object/PRJNA739515?reviewer=jqgj6n7reasr8ei9a20riogqok).

## Abbreviations

ADSCs: Adipose-derived stem cells
ALP: Alkaline phosphatase
ARS: Alizarin Red S
DEGs: Differentially expressed genes
dhASCs: Osteogenically differentiated hASCs
DMEGs: Differentially methylated and expressed genes
DMGs: Differentially methylated genes
GO: Gene Ontology
GSEA: Gene set enrichment analysis
hASCs: Human adipose-derived stem cells
hBMSCs: Human bone marrow-derived mesenchymal stem cells
KEGG: Kyoto Encyclopedia of Genes and Genomes
m^6^A: N^6^-methyladenosine
m^6^A MeRIP-Seq: Methylated RNA immunoprecipitation and sequencing
PPI: Protein-protein interaction
qRT-PCR: Quantitative real-time polymerase chain reaction
RNA-seq: High-throughput sequencing for RNA
uhASCs: Undifferentiated hASCs
UTR: Untranslated region
