# Supplementary figures and images for: Transcriptome-wide m6A methylome during osteogenic differentiation of human adipose-derived stem cells
Source: Stem Cell Res Ther. 2021 Sep 1;12:489. doi: 10.1186/s13287-021-02508-1 (PMC8411547; doi:10.1186/s13287-021-02508-1)

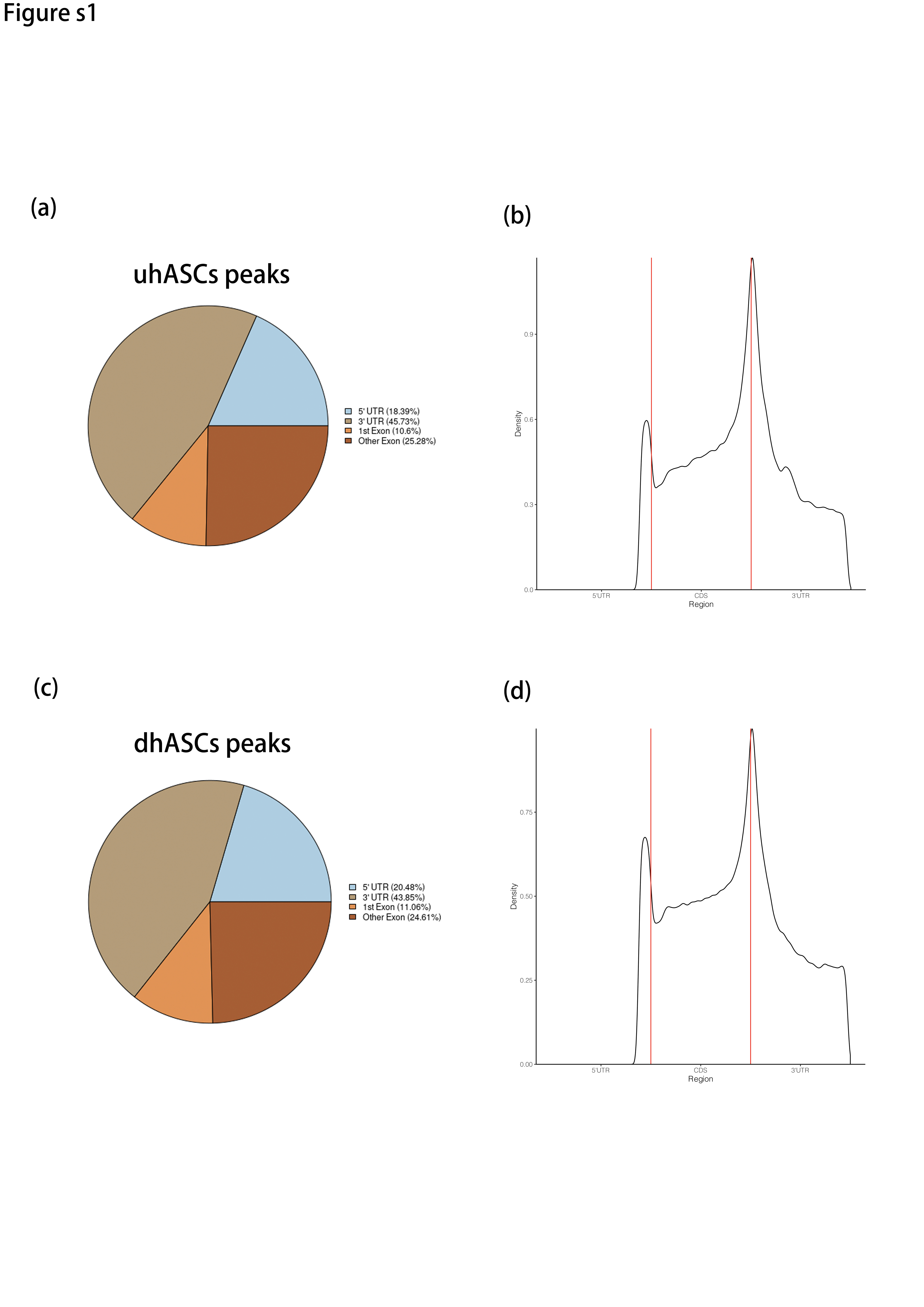

Supplement: Supplementary file 1 — Additional file 1: Figure S1. m6A peak calling for uhASCs and dhASCs. a Pie charts showing distribution of m6A peaks of uhASCs in different gene context. b Accumulation of m6A peaks of uhASCs along transcripts. c Pie charts showing distribution of m6A peaks of dhASCs in different gene context. d Accumulation of m6A peaks of dhASCs along transcripts. hASCs, human adipose-derived stem cells; uhASCs, undifferentiated hASCs; dhASCs, osteogenically differentiated hASCs. [file 13287_2021_2508_MOESM1_ESM.jpg]

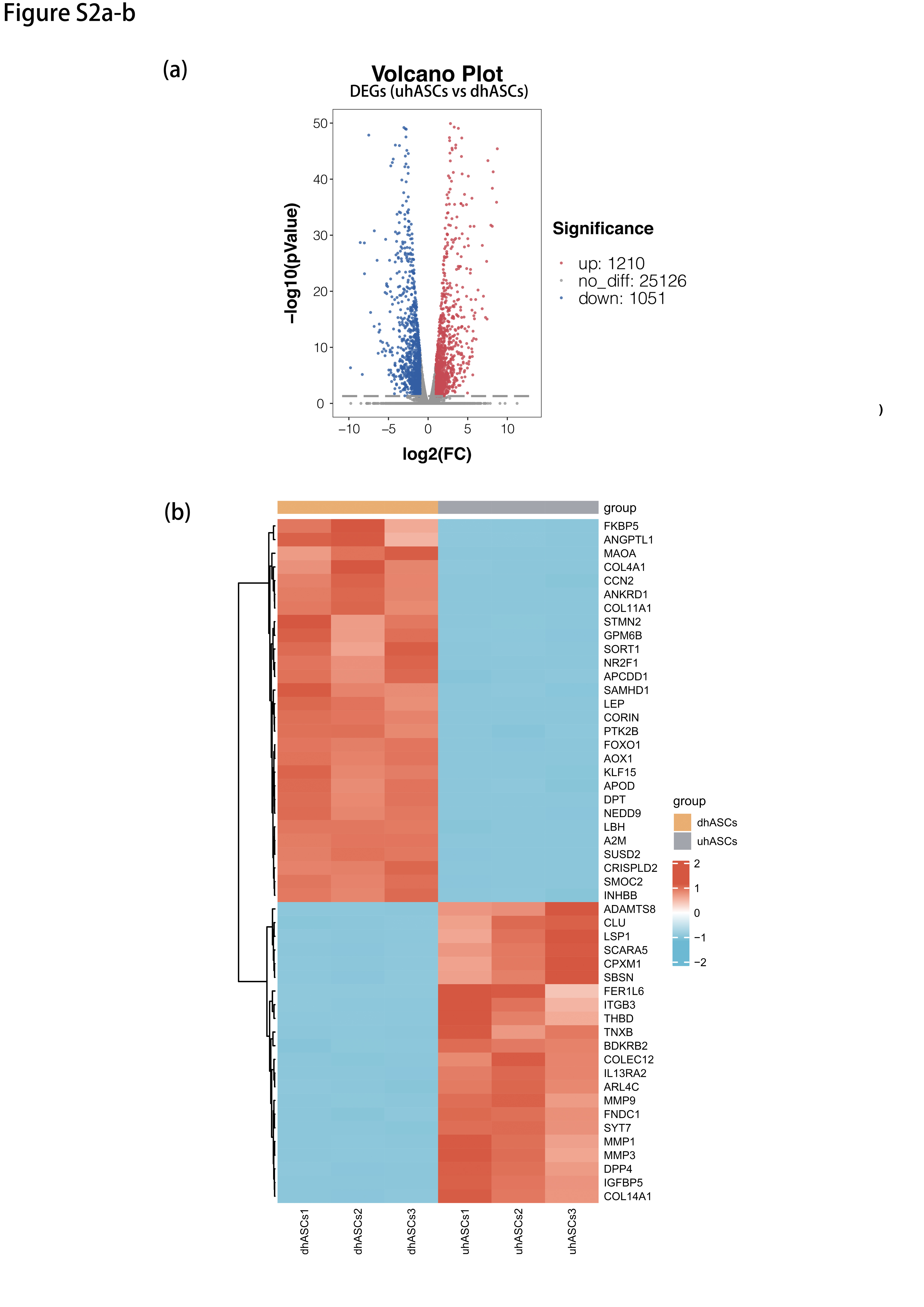

Supplement: Supplementary file 2 — Additional file 2: Figure S2. Gene expression profile during osteogenesis of hASCs by RNA-seq. a Volcano plots displaying the DEGs (fold change ≥ 2 and p < 0.05). b Hierarchical clustering analysis of the top 50 DEGs (ranking by p value). c GO enrichment analysis for upregulated genes. d KEGG pathway analysis for upregulated genes. e GO enrichment analysis for downregulated genes. f KEGG pathway analysis for downregulated genes. DEGs, differentially expressed genes; hASCs, human adipose-derived stem cells; uhASCs, undifferentiated hASCs; dhASCs, osteogenically differentiated hASCs; GO, gene ontology; KEGG, Kyoto Encyclopedia of Genes and Genomes; FC, fold change. [file 13287_2021_2508_MOESM2_ESM.zip › Supplementary Figure S2a-b.jpg]

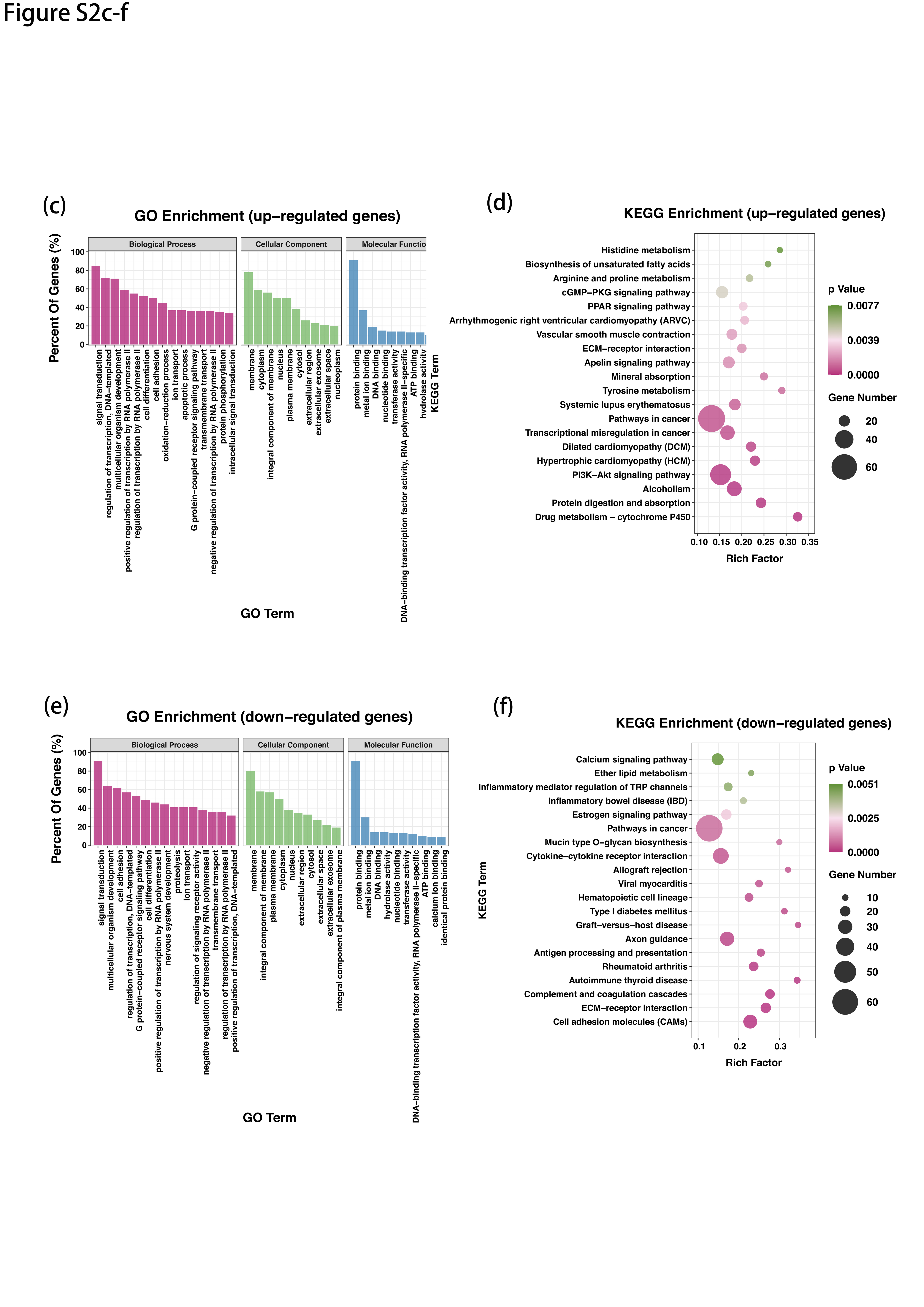

Supplement: Supplementary file 2 — Additional file 2: Figure S2. Gene expression profile during osteogenesis of hASCs by RNA-seq. a Volcano plots displaying the DEGs (fold change ≥ 2 and p < 0.05). b Hierarchical clustering analysis of the top 50 DEGs (ranking by p value). c GO enrichment analysis for upregulated genes. d KEGG pathway analysis for upregulated genes. e GO enrichment analysis for downregulated genes. f KEGG pathway analysis for downregulated genes. DEGs, differentially expressed genes; hASCs, human adipose-derived stem cells; uhASCs, undifferentiated hASCs; dhASCs, osteogenically differentiated hASCs; GO, gene ontology; KEGG, Kyoto Encyclopedia of Genes and Genomes; FC, fold change. [file 13287_2021_2508_MOESM2_ESM.zip › Supplementary Figure S2c-f.jpg]
