# Supplementary material for: Transcriptome-wide m6A methylome during osteogenic differentiation of human adipose-derived stem cells
Source: Stem Cell Res Ther. 2021 Sep 1;12:489. doi: 10.1186/s13287-021-02508-1 (PMC8411547; doi:10.1186/s13287-021-02508-1)
Supplement: Supplementary file 3 — Additional file 3: Table S1. Primers used for qRT-PCR. [file 13287_2021_2508_MOESM3_ESM.docx]

Supplementary Table S1. Primers used for quantitative real-time-PCR.

| Gene | Forward Primer | Reverse Primer |
| --- | --- | --- |
| GAPDH | GTCTCCTCTGACTTCAACAGCG | ACCACCCTGTTGCTGTAGCCAA |
| METTL3 | CTATCTCCTGGCACTCGCAAGA | GCTTGAACCGTGCAACCACATC |
| METTL14 | CTGAAAGTGCCGACAGCATTGG | CTCTCCTTCATCCAGATACTTACG |
| FTO | CCAGAACCTGAGGAGAGAATGG | CGATGTCTGTGAGGTCAAACGG |
| ALKBH5 | CCAGCTATGCTTCAGATCGCCT | GGTTCTCTTCCTTGTCCATCTCC |
| ALPL | GCTGTAAGGACATCGCCTACCA | CCTGGCTTTCTCGTCACTCTCA |
| RUNX2 | CCCAGTATGAGAGTAGGTGTCC | GGGTAAGACTGGTCATAGGACC |
| BGLAP | CGCTACCTGTATCAATGGCTGG | CTCCTGAAAGCCGATGTGGTCA |
| SPP1 | CGAGGTGATAGTGTGGTTTATGG | GCACCATTCAACTCCTCGCTTTC |
| SP7 | TTCTGCGGCAAGAGGTTCACTC | GTGTTTGCTCAGGTGGTCGCTT |
| COL1A1 | GATTCCCTGGACCTAAAGGTGC | AGCCTCTCCATCTTTGCCAGCA |
| VEGFA | TTGCCTTGCTGCTCTACCTCCA | GATGGCAGTAGCTGCGCTGATA |
| CD44 | CCAGAAGGAACAGTGGTTTGGC | ACTGTCCTCTGGGCTTGGTGTT |
| MMP2 | AGCGAGTGGATGCCGCCTTTAA | CATTCCAGGCATCTGCGATGAG |
| HGF | GAGAGTTGGGTTCTTACTGCACG | CTCATCTCCTCTTCCGTGGACA |
| SPARC | TGCCTGATGAGACAGAGGTGGT | CTTCGGTTTCCTCTGCACCATC |
